# Characterization and Comparative Analyses of Muscle Transcriptomes in Dorper and Small-Tailed Han Sheep Using RNA-Seq Technique

**DOI:** 10.1371/journal.pone.0072686

**Published:** 2013-08-30

**Authors:** Chunlan Zhang, Guizhi Wang, Jianmin Wang, Zhibin Ji, Zhaohuan Liu, Xiushuang Pi, Cunxian Chen

**Affiliations:** Shandong Provincial Key Laboratory of Animal Biotechnology and Disease Control and Prevention, College of Animal Science and Veterinary Medicine, Shandong Agricultural University, Taian, Shandong Province, China; University of Sydney, Australia

## Abstract

The sheep is an important domestic animal and model for many types of medically relevant research. An investigation of gene expression in ovine muscle would significantly advance our understanding of muscle growth. RNA-seq is a recently developed analytical approach for transcriptome profiling via high-throughput sequencing. Although RNA-seq has been recently applied to a wide variety of organisms, few RNA-seq studies have been conducted in livestock, particularly in sheep. In this study, two cDNA libraries were constructed from the biceps brachii of one Small-tailed Han sheep (SH) and one Dorper sheep (DP). The Illumina high-throughput sequencing technique and bioinformatics were used to determine transcript abundances and characteristics. For the SH and DP libraries, we obtained a total of 50,264,608 and 52,794,216 high quality reads, respectively. Approximately two-thirds of the reads could be mapped to the sheep genome. In addition, 40,481 and 38,851 potential coding single nucleotide polymorphisms (cSNPs) were observed, respectively, of which a total of 59,139 cSNP coordinates were different between the two samples. Up to 5,116 and 5,265 respective reference genes had undergone 13,827 and 15,684 alternative splicing events. A total of 6,989 reference genes were extended at the 5’, 3’ or both ends, and 123,678 novel transcript units were found. A total of 1,300 significantly differentially expressed genes were identified between the two libraries. These results suggest that there are many differences in the muscle transcriptomes between these two animals. This study addresses a preliminary analysis and offers a foundation for future genomic research in the sheep.

## Introduction

As a predominant domestic animal, the sheep (*Ovis. aries*) not only provides meat, milk, wool, cashmere and fur but is also an important model organism for cardiology research [1,2], reproductive medicine [3], respiratory medicine [4,5] and many other fields. Understanding the genetic principles of meat traits would be helpful for promoting sheep growth and for basic biology and human medicine.

Over the past decade, microarray hybridization has been commonly used to measure gene expression [6–10]. Using the first specialized transcriptome-wide sheep DNA oligonucleotide microarray, the gene expression profiles of the developing fetal ovine longissimus dorsa of the Texel sheep and Ujumqin sheep were investigated [11]. More recently, massive parallel sequencing has played a primary role in quantifying gene expression by deep sequencing of the transcriptome. Also known as RNA-seq, deep sequencing provides comprehensive data for understanding gene structures and expression patterns. Deep sequencing the transcriptome allows not only understanding gene expression models but also the discovery of new transcripts, single nucleotide polymorphisms (SNPs), alternative splicing (AS) events and other information. Recently, the efficacy of RNA-seq has been demonstrated in many organisms, including humans [12], pigs [13] and bovines [14], and model organisms, including yeast [15], *Arabidopsis* [16], and mice [17].

Until now, reports on the sheep transcriptome that have applied RNA-seq technology are very limited. Although many novel transcripts and differentially expressed genes have been published for sheep bone [18], no global transcriptome analyses of sheep muscle using RNA-seq have been performed. Dorper sheep (DP) are a foreign breed with two types of black and white head. Their legs are short and thin, and they have a flat back and wide waist. Because of their rapid muscle growth, DP is world-famous as a meat-producing breed. While the indigenous Chinese breed, the Small-tailed Han sheep (SH), with long, strong limbs and an elliptical fan-shaped tail, has a slower growth rate and is a dual-purpose breed [19]. To gain a better understanding of muscle gene expression, we performed RNA-seq using the Illumina system to analyze and compare the two sheep. Using bioinformatic analysis, we characterized the sheep muscle transcriptome and analyzed AS, coding single nucleotide polymorphisms (cSNPs) and differentially expressed genes. These results provide basic data for future studies.

## Materials and Methods

### Ethics Statement

All animal experiments were approved by the Institutional Animal Care and Use Ethics Committee of Shandong Agricultural University (Permit Number: 2004006) and performed in accordance with the “Guidelines for Experimental Animals” of the Ministry of Science and Technology (Beijing, China). All surgery was performed according to recommendations proposed by the European Commission (1997), and all efforts were made to minimize suffering.

### Animal and Muscle Tissue Collection

In this study, one female Small-tailed Han sheep and one female DP, each aged 11 months, were selected from two purebred herds. The appearance and shape of the sheep completely conformed to their varietal characteristics [19]. Their body conditions were healthy, and their weights were moderate. The sheep were fed in stables. The room temperature was uncontrolled, and the environment was exposed to natural lighting. The animals were slaughtered quickly to collect approximately 3 g of biceps brachii. The fresh tissues was immediately frozen on liquid nitrogen after collection and then stored at -80°C for preservation until use.

### RNA Extraction and Quality Assessment

Total RNA was isolated from each tissue using the TRIzol reagent according to the manufacturer’s protocol (Invitrogen, Burlington, ON, Canada). DNA was removed from RNA extracts by incubation with RNase-free DNaseⅠ(New England Biolabs) for 30 min at 37°C. The RNA integrity and concentration was evaluated using an Agilent Technologies 2100 Bioanalyzer. Both samples had RNA Integrity Number (RIN) values greater than 7.5.

### cDNA Library Construction and Illumina Sequencing

Poly(A) mRNA was isolated from total RNA using oligo(dT) magnetic beads (Invitrogen, Carlsbad, CA, USA). Fragmentation buffer was added to break the purified mRNA into short fragments. Using these short fragments as templates, first-strand cDNA synthesis was performed using random hexamer primers and reverse transcriptase (Invitrogen). Second-strand cDNA was synthesized using RNase H (Invitrogen), DNA polymerase I (New England Biolabs), dNTPs and buffer. Subsequently, the short fragments were purified using the QIAquick PCR extraction kit, eluted with EB buffer and end repaired. Poly(A) tails were added, and the fragments were ligated to sequencing adaptors. We then selected suitable fragments as templates for PCR amplification according to the results of agarose gel electrophoresis. The average insert size for the paired-end libraries was 200 bp (from 180 to 220 bp). Two paired-end cDNA libraries were constructed, one for each of the two samples (SH muscle and DP muscle). Finally, the cDNA libraries were loaded onto the flow cell channels of an Illumina HiSeq^TM^ 2000 platform for paired-end 90 bp×2 sequencing at the Beijing Genomics Institute (BGI), Shenzhen, China. We deposited our sequencing dataset in the NCBI GEO (Gene Expression Omnibus) repository as “Next Generation Sequencing of Different Ovine Muscle’s Transcriptomes”. The accession number is GSE43316.

### Read Mapping to the Reference Genome and Sequencing Quality Assessment

The *O. aries* genome was downloaded (http://www.livestockgenomics.csiro.au/sheep/oar2.0.php), and 20,236 reference genes were downloaded (http://www.livestockgenomics.csiro.au/sheep/sheep.v4.gff.filter.chr.annot.gff3.gz). After discarding low-quality raw reads, including sequencing adapters, and rejecting reads with more than 5% unknown nucleotides or low quality sequence (more than half of the base qualities less than 5), high-quality reads were obtained. The high-quality reads were mapped to the *O. aries* genome, and gene sequences were annotated using SOAP2 [20]. No more than m base mismatches (the m default is 5) were included in the alignment. Unmapped or multi-position matched reads were excluded from further analyses. The proportions of high-quality reads that mapped to the genome and to genes provided an overall assessment of the sequencing quality.

The sequencing quality assessment was performed by a sequencing randomness assessment of the mRNA/cDNA relative to the known reference genes and according to the distribution of reads along the reference genome. Because the reference genes were of different lengths, reads were located at positions relative to the reference genes according to the ratios of the reads’ locations to the reference gene lengths. Subsequently, the total number of reads aligned to reference genes was counted. The read distribution among the reference genes would be homogeneous if the fragmentation was random. After reviewing the gene distribution resulting from different mRNA and cDNA fragmentation strategies [21], we chose to use RNA fragmentation in our experiments, which had a more even read distribution. The read distribution relative to the reference genome provided their specific locations, including their relationships to exon, intron and intergenic regions.

### Analysis of Gene Expression and Annotation

To analyze gene expression, we calculated gene coverage and gene expression levels. Gene coverage is the percentage of a gene covered by reads. This value is equal to the ratio of the number of bases in a gene covered by uniquely mapped reads to the total number of bases in that gene. The reads per kilobase per million mapped reads (PRKM) method, which measures the numbers of reads per kilobase of an exon region in a gene per one million mapped reads, was used for the gene expression level calculation [17]. The formula is as follows:

$$RPKM=\frac{C}{NL}\times 10^{9}$$

Where RPKM (A) is the expression level of gene A, C is the number of reads that are uniquely aligned to gene A, N is the total number of reads that is uniquely aligned to all genes, and L is the number of bases in the CDS of gene A. The RPKM method is able to control for differences in gene length and sequencing discrepancies in the calculation of gene expression levels. Therefore, the calculated gene expression level can be used for directly comparing gene expression levels between samples. If there was more than one transcript for a gene, the longest transcript was used to calculate its expression level and coverage.

### Differentially Expressed Gene Analysis

Differentially expressed genes and their corresponding P-values were determined using methods described by Audic and Claverie [22]. The false discovery rate (FDR) was used to assess the P-value in multiple tests. Fold changes (log _2_Ratio) were estimated according to the normalized gene expression level in each sample [23,24]. We used FDR ≤ 0.001 and the absolute value of log _2_Ratio ≥1” as the threshold to judge significance differences in gene expressing.

### Identification of Novel Transcript Units

Because current databases may be incomplete, novel transcript units (TUs) may discovered. In this study, transcript units found in intergenic regions farther than 200 bp away from known genes with a continuous mapping length ≥150 bp and average coverage ≥ 2 were considered putative novel TUs.

### cSNPs Analysis

We detected cSNPs using SOAPsnp [25]. SOAPsnp is a member of SOAP (short oligonucleotide analysis package). The program is a resequencing utility that can assemble a consensus sequence for the genome of a newly sequenced individual based on alignment of raw sequencing reads to a known reference. Through comparison with the reference genome, cSNPs can be identified in the consensus sequence.

Based on the alignment of short reads to the reference genome together with their corresponding sequencing quality scores, we inferred the genotype with highest posterior probability at each site using Bayes’ theorem (the reverse probability model). Thus, we considered the intrinsic bias and errors that are common in Illumina/Solexa sequencing data and recalibrated the quality values to infer the consensus sequence.

### Identification of AS

**Figure 1:**
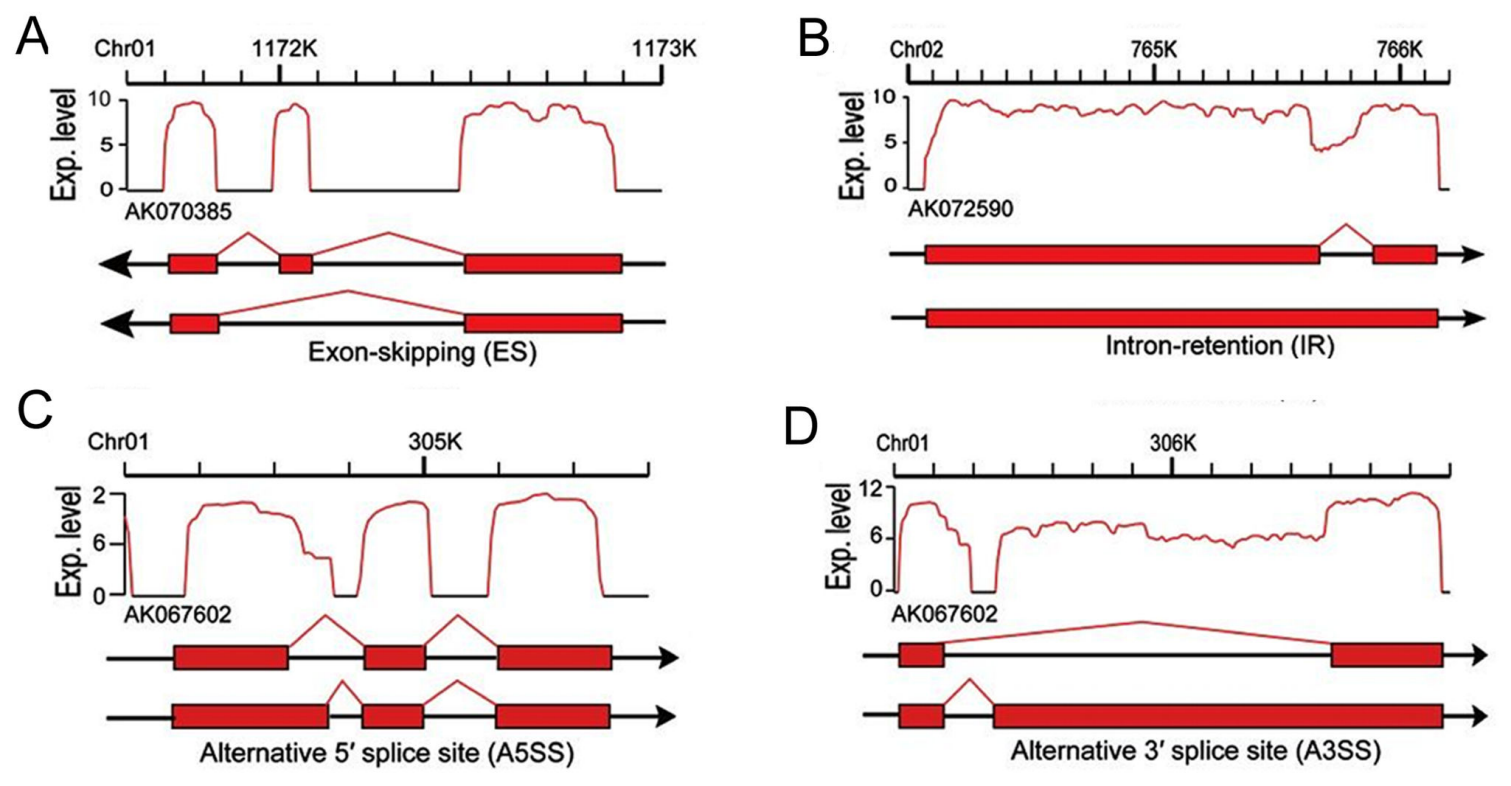
Sketch of alternative splicing in the ovine transcriptome. The schematic depicts the four primary types of AS events in the ovine transcriptome. The red curve indicates expression level (log_2_ of the RPKM value). The red bar denotes the exon, and the red line shows the linkage between two exons.

Recently, there have been many new AS events discovered in humans [26], *Arabidopsis* [27], rice [28] and other organisms. There are seven main types of AS events: exon skipping (ES), intron retention (IR), alternative 3’ splice sites (A3SS), alternative 5’ splice sites (A5SS), alternative first exon (AFE), alternative last exon (ALE) and mutually exclusive exon (MEE) [29]. Because the last three AS event types were not included in this study due to high false positive rates with the version of the analysis program we utilized, we analyzed four known types of AS models: A3SS, A5SS, ES and IR. Figure 1 summarizes the four types of AS detected by the SOAPsplice program.

We identified sequence reads that mapped to the regions of computationally determined theoretical splicing junctions. To detect AS events, we first detected junction sites, which provided information about intron/exon boundaries, and then detected combinations of different exons in a transcript using SOAPsplice. Next, all junction sites of the same gene were used to distinguish the different transcript types due to AS.

### Optimization of Gene Boundary Annotations

**Figure 2:**
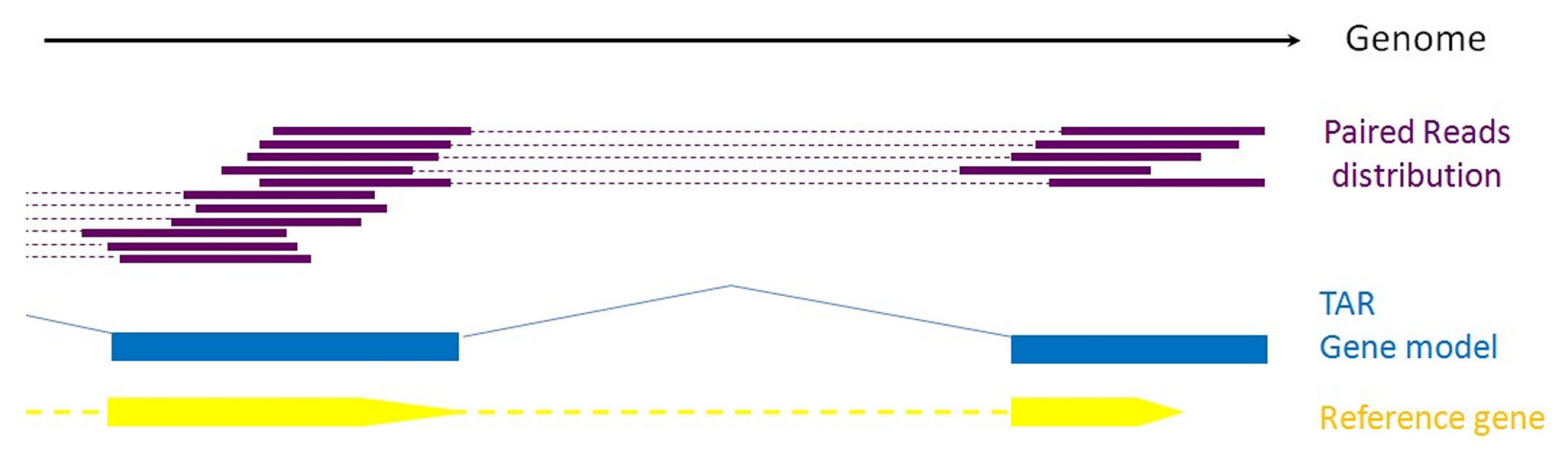
Gene boundary-refining algorithm sketch. This diagram illustrates the gene extension process. Purple lines indicate the continuous and overlapping regions of sequence similarity between paired sequencing reads and the genome. The blue thick lines denote transcriptionally active regions (TARs) identified by overlapping reads. Different TARs were connected using the thin blue lines to form potential gene models. Through comparisons of the existing yellow reference gene with the gene model, the annotated gene could be extended at the 5’ end, the 3’ end or at both ends.

We optimized the annotated gene boundaries according to the distribution of the RNA-seq reads, information from paired-end sequences and *homologous* reference genes annotations. After read alignment to the reference genome, we inferred the genomic read distribution by aligning continuous and overlapping reads in a transcriptionally active region (TAR). Using the paired-end data, we connected sets of different TARs to form potential gene models. By comparing potential gene models with the existing gene annotations, we extended the 5’ and 3’ gene boundaries. Figure 2 illustrates a 3’ extension.

## Results

### Sequencing Data Summary

In this study, we constructed two cDNA sequencing libraries using muscles from SH and DP. The libraries were sequenced using a High-seq 2000 sequencing platform at BGI-Shenzhen, China, and two sets of reads were obtained. The sequencing reads were submitted to the NCBI GEO (Gene Expression Omnibus) database under accession number GSE43316. The total read length was 9.27 gigabases (Gb), more than three-fold the sheep genome size. Altogether, we acquired more than 103 megabases (Mb) of paired-end high-quality reads for the two samples. All of the reads were 90 bp. These data were preliminarily analyzed according to the Beijing Genomics Institute (BGI) bioinformatics protocols for RNA-seq. We aligned all high-quality short reads onto the reference genome (ISGC, *O. aries* genome v2.0) and known reference genes (approximately 20,236 terms). As shown in Tables 1 and 2, more than two-thirds of the reads could be mapped to the sheep genome (DP, with 71.82% alignment, was slightly higher than SH, with 68.50%), but a little more than one-third of each sample could be mapped to the reference genes (DP, with 33.10% reference gene mapping, was lower than SH, with 42.77%). Nearly half of the reads could be perfectly matched to the genome, but less than one-third could be perfectly matched to the reference genes. In addition, more than half of the mapped reads had unique genomic locations, but only 40.45% and 31.55% of the reads corresponded to reference genes. Unfortunately, of all of the high-quality reads, approximately 60% could not be mapped to reference genes.

**Table 1:** A summary of the sequencing reads alignment to the *Ovis aries* genome.

Sample	SH	DP
Total reads	50,264,608	52,794,216
Total base-pairs	4,523,814,720	4,751,479,440
Total mapped reads	34,205,468 (68.05%)	37,916,265 (71.82%)
Perfect matched reads	23,585,449 (46.92%)	26,897,927 (50.95%)
<=5 bp mismatched reads	10,620,019 (21.13%)	11,018,338 (20.87%)
Unique matched reads	29,083,338 (57.86%)	29,378,191 (55.65%)
Multi-position matched reads	5,122,130 (10.19%)	8,538,074 (16.17%)
Fragments mapped to Exons	10,123,649 (29.60%)	8,319,089 (21.94%)
Fragments mapped to Introns	917,892 (2.68%)	921,292 (2.43%)
Fragments mapped to Intergenic	18,963,691 (55.44%)	25,177,123 (66.40%)
Reads overlapped with exons	4,200,236 (12.28%)	3,498,761 (9.23%)
Total unmapped reads	16,059,140 (31.95%)	14,877,951 (28.18%)
Highly difference expressed genes (≥500 RPKM)	24 (1.85%)	17 (1.31%)
Medium difference expressed genes (≥10 to 500 RPKM))	409 (31.46%)	362 (27.85%)
Low difference expressed genes (<10 RPKM)	867 (66.69%)	921 (70.85%)

**Table 2:** A summary of the sequencing reads alignment to the reference genes.

Sample	SH	DP
Total reads	50,264,608	52,794,216
Total base-pairs	4,523,814,720	4,751,479,440
Total mapped reads	21,500,207 (42.77%)	17,474,062 (33.10%)
Perfect matched reads	15,965,223 (31.76%)	13,009,047 (24.64%)
<=4 bp mismatched reads	5,534,984 (11.01%)	4,465,015 (8.46%)
Unique matched reads	20,329,585 (40.45%)	16,656,621 (31.55%)
Multi-position matched reads	1,170,622 (2.33%)	817,441 (1.56%)
Total unmapped reads	28,764,401 (57.23%)	35,320,154 (66.90%)

### Sequencing Quality Assessment

**Figure 3:**
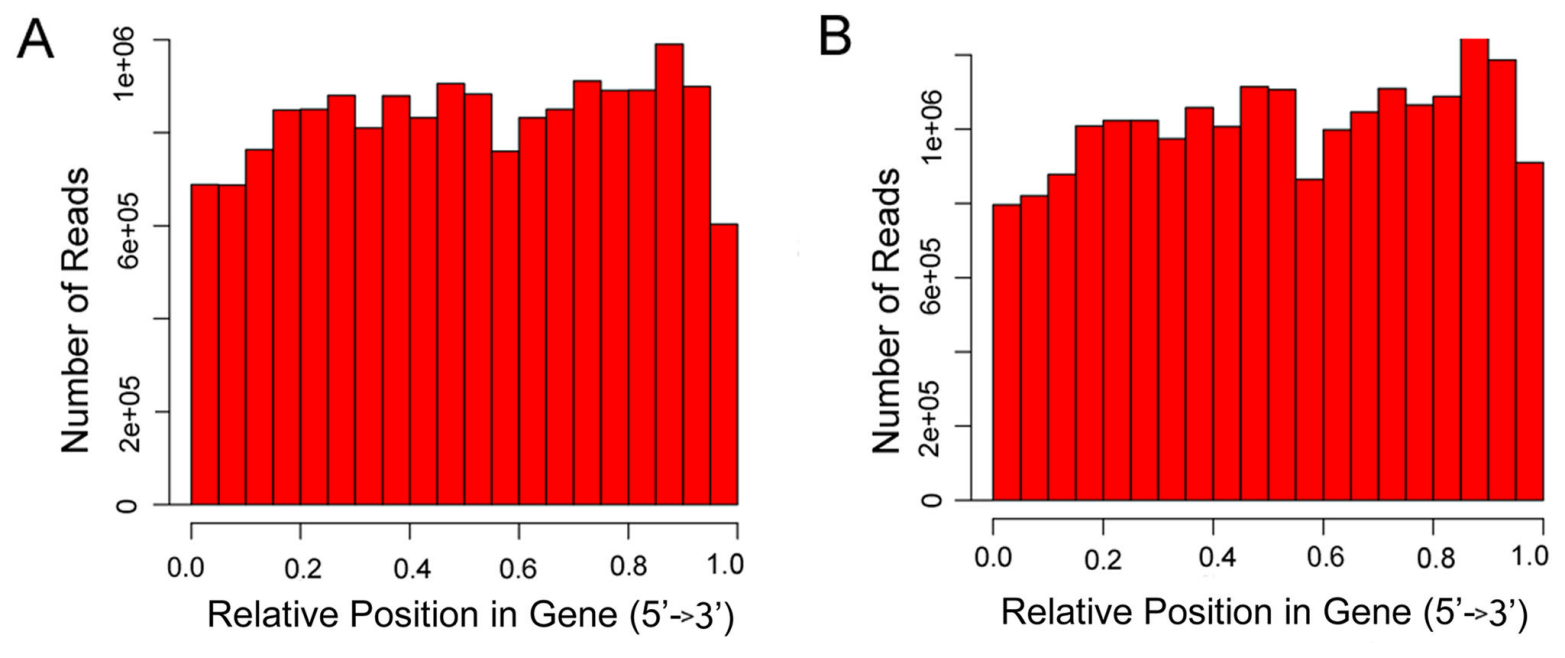
Distribution of reads mapped to the reference genes. Each reference gene is oriented from the 5’ to 3’ end as 0.0 to 1.0. (A) shows the number of reads in the relation to the reference genes in the Dorper sheep (DP) library, and (B) shows the same for the Small-tailed Han sheep (SH) library. The reads are evenly distributed in from the 5’ to 3’ end in the reference genes in the two samples.

Fragmentation of mRNA/cDNA was performed using physical or chemical methods during the experimental phase of the transcriptome analysis. If the fragmentation randomness was poor, reads would be more frequently generated from specific regions of the original transcripts, and the ensuing analysis would be negatively affected. We analyzed the read distribution mapped to reference genes (Figure 3). From this analysis, we observed that the reads were evenly distributed over the genes from 5’ to 3’. To further understand the sheep genome read distribution, we statistically examined the total read distribution. For all the mapped reads from the DP and SH libraries, 21.94% and 29.60% fragments were matched to annotated exons, 2.43% and 2.68% were located in introns, 9.23% and 12.28% overlapped exons, and the remaining 66.40% and 55.44% were assigned to intergenic regions (Table 1), respectively. We concluded that the reads could cover the reference genome and genes.

### Gene Expression and Annotation

**Figure 4:**
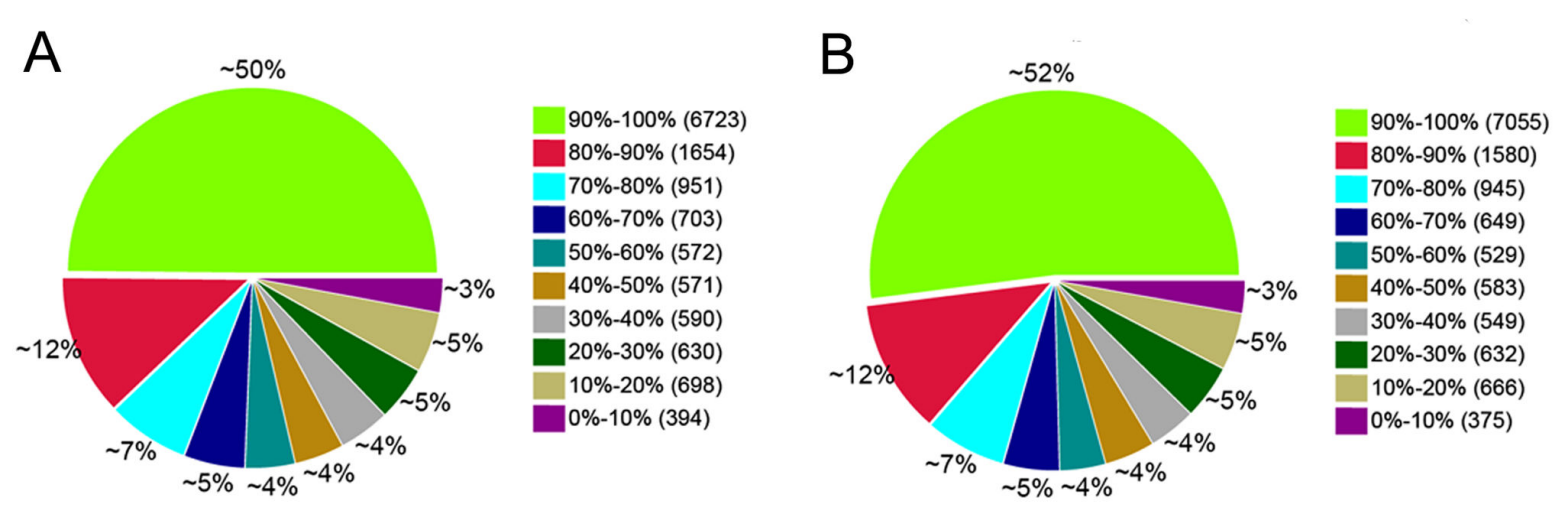
Distribution of genic coverage in the two ovine transcriptomes. **A**, **DP; B, SH**. Gene coverage is the percentage of a gene covered by reads. This value is equal to the ratio of total base count in a gene covered by uniquely mapped reads to the total base count for that gene. The read distribution is similar in both RNA-seq libraries, with approximately 50% of the reference genes having 90%-100% coverage.

RNA-seq analysis of the two types ovine muscle revealed extensive gene expression. We evaluated gene expression levels by counting the number of RPKM (Table S1). In the two libraries, there were 13,488 (66.65%) and 13,563 (67.02%) known reference genes expressed in the DP and SH samples, respectively, and 12,761 (47.17%) genes were identical between the two samples. As shown in Table 3, less than 1% of the genes were expressed at more than 1,000 RPKM; approximately 5.5% were expressed between 100–1,000 RPKM, and 94% of the genes were expressed at less than 100 RPKM. The maximum expression level of an annotated gene was 51,132 and 51,099 RPKM in the DP and SH analyses, respectively. As shown in Figure 4, there was a similar read distribution in the two RNA-seq libraries. Between the DP and SH libraries, 50% and 52% of the reference genes had 90-100% coverage, respectively, and 12% of the annotated genes had 80-90% coverage.

**Table 3:** Gene expression and annotation by RPKM.

RPKM	Gene number of DP (%)	Gene number of SH (%)
0-100	12,618 (93.55%)	12,746 (93.98%)
100-1,000	768 (5.69%)	735 (5.42%)
1,000-10,000	90 (0.67%)	72 (0.53%)
>10,000	12 (0.09%)	10 (0.07%)
Total	13,488	13,563

### Differentially Expressed Genes (DEGs) Analysis

**Figure 5:**
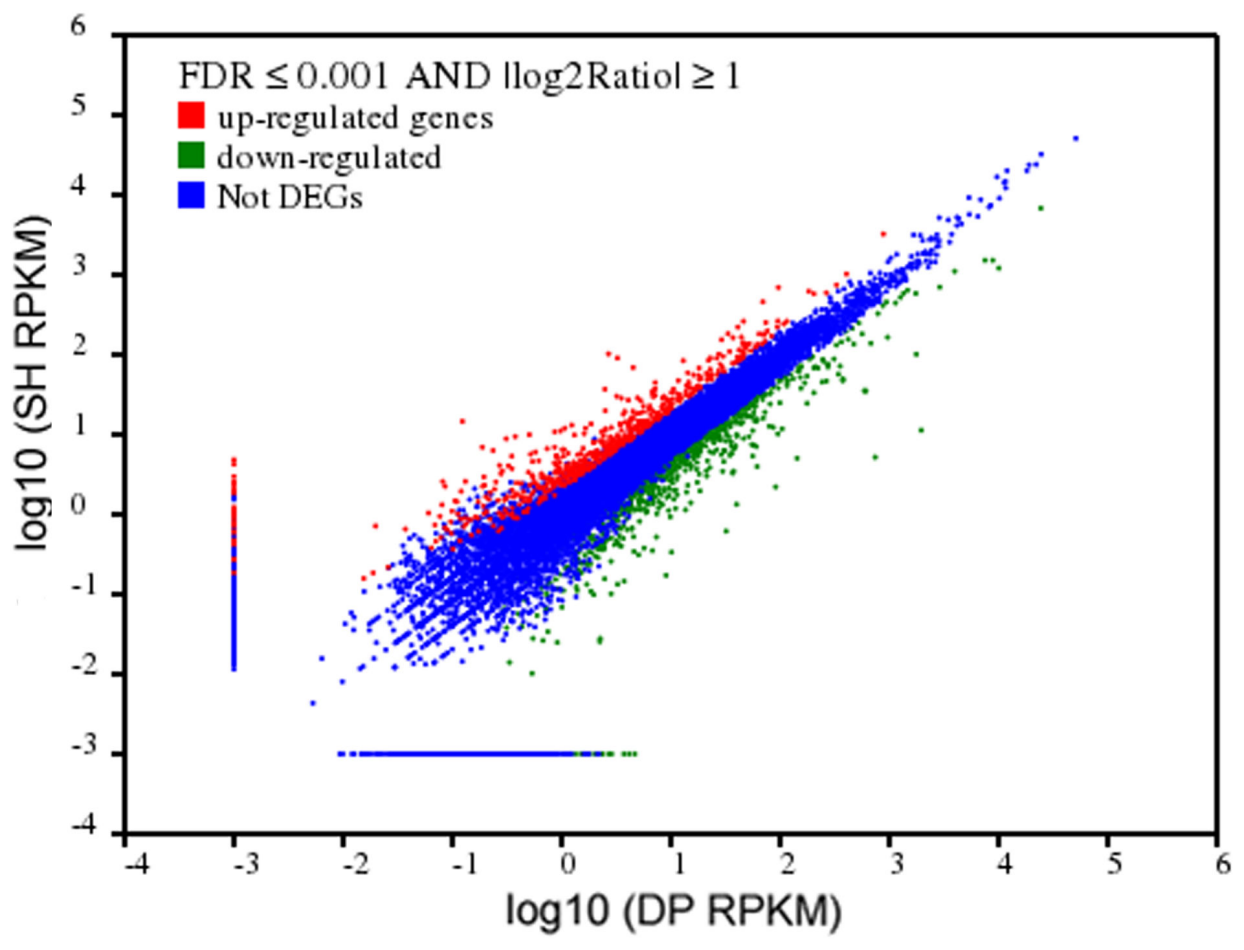
Comparison of gene expression levels between the two libraries. The X and Y-axes show the mRNA expression levels in the two samples. The up-regulated and down-regulated genes are shown in red and green, respectively. Blue dots represent genes with similar expression level.

Deep RNA sequencing in the two muscle types allowed us to investigate differentially transcription. Using FDR ≤0.001 and the absolute value of the log _2_Ratio ≥1 as the threshold values, 1,300 genes were found to be differentially expressed, with 554 genes up-regulated and 746 genes down-regulated in the SH library (Table S2 and Figure 5). We found 27 genes that were only expressed in the SH library and 36 genes only in the DP library. The 1,300 DEGs could be categorized into three groups: highly (≥500 RPKM), medium (≥10 to 500 RPKM) and low (<10 RPKM) expressed genes (Table 1). Comparing the two libraries, there were slightly more highly and medium differentially expressed SH than DP genes.

### Identification of Novel Transcript Units

Based on the alignment of the sequencing data to the sheep reference genome, we obtained 66,668 and 57,010 novel TUs in the DP and SH libraries, respectively. The mean novel TU length was 343 bp, with a size range from 150 to 52,905 bp. In the DP and SH libraries, approximately 66.87% and 66.10% of novel TUs had > 1 exons, and the longest TU contained 194 and 178 exons, respectively (Table S3). In additional, we found many repeated or incompletely repeated TUs between the two samples.

### cSNPs Exploitation

**Figure 6:**
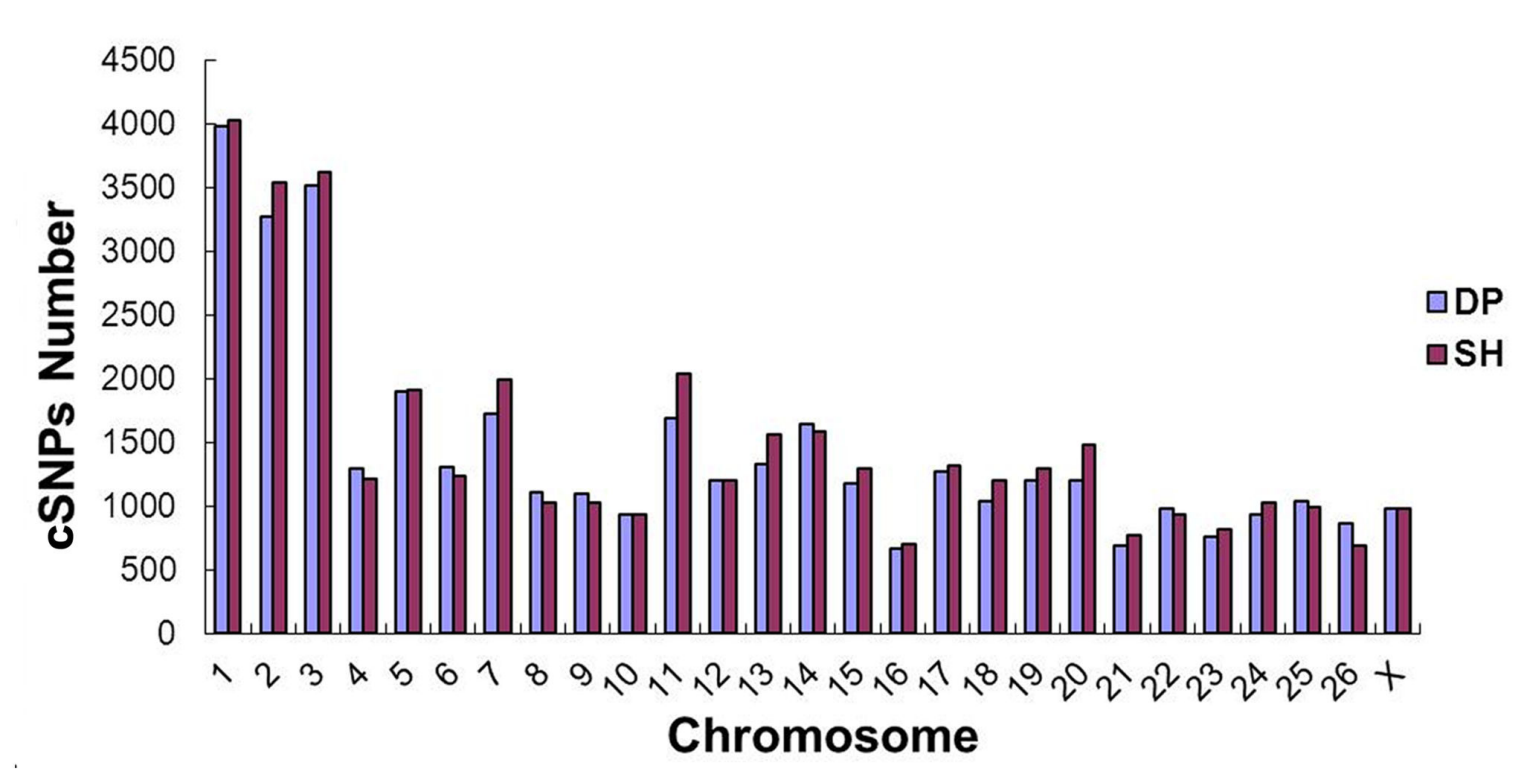
The distribution of cSNPs in the *Ovis aries* genome. The distribution of 79,332 putative cSNPs on the 26 sheep autosomes and the X allosome according to the two sequencing datasets are shown. All of the cSNPs distributed primarily on chromosomes 1, 2 and 3.

By aligning transcript sequences to the reference genome sequences, we identified candidate cSNPs. We found 38,851 and 40,481 potential cSNPs in the DP and SH analyses, respectively (Table S4). The most common change was A/G, followed by T/C and C/T (Table 4). In addition, we found 59,139 cSNPs coordinates with different locations between the two samples (20,288 in the DP and 38,851 in the SH sample), and 5,504 cSNPs were identical. Next, we mapped all cSNPs and the observed different cSNPs between the two samples on the 27 sheep chromosomes (26 autosomes and 1 allosome). The two libraries had similar mapping results, with all of the cSNPs distributed primarily on chromosomes 1, 2 and 3. However, the number of cSNPs was slightly higher in SH than DP on the three chromosomes (Figure 6). The observed differential cSNPs were scattered unevenly on each chromosome from 5’ to 3’ and were relatively concentrated in certain areas of the chromosomes. For example, the different cSNPs were clustered on a 80-120 Mb region of the chromosome 1. Between the two libraries, the number of different cSNPs was obvious in some chromosomal regions. For example, there were obviously more DP cSNPs in a 20-25 Mb region of chromosome 23 than SH cSNPs (Figure S1).

**Table 4:** A summary of the cSNPs by nucleotide change.

Allele variation	DP	SH	Allele variation	DP	SH
A→G	5,286	4,984	A→C	818	764
T→C	5,034	4,723	T→G	814	776
C→T	3,908	3,483	C→S	530	604
G→A	3,834	3,433	T→A	527	494
C→Y	3,292	3,949	C→M	511	588
G→R	3,176	3,994	A→T	489	468
A→R	2,428	3,009	G→S	467	594
T→Y	2,395	3,046	A→M	456	539
G→C	892	843	G→K	446	554
C→G	888	842	T→K	434	535
C→A	862	826	T→W	275	363
G→T	825	766	A→W	255	303

### AS Analysis

**Figure 7:**
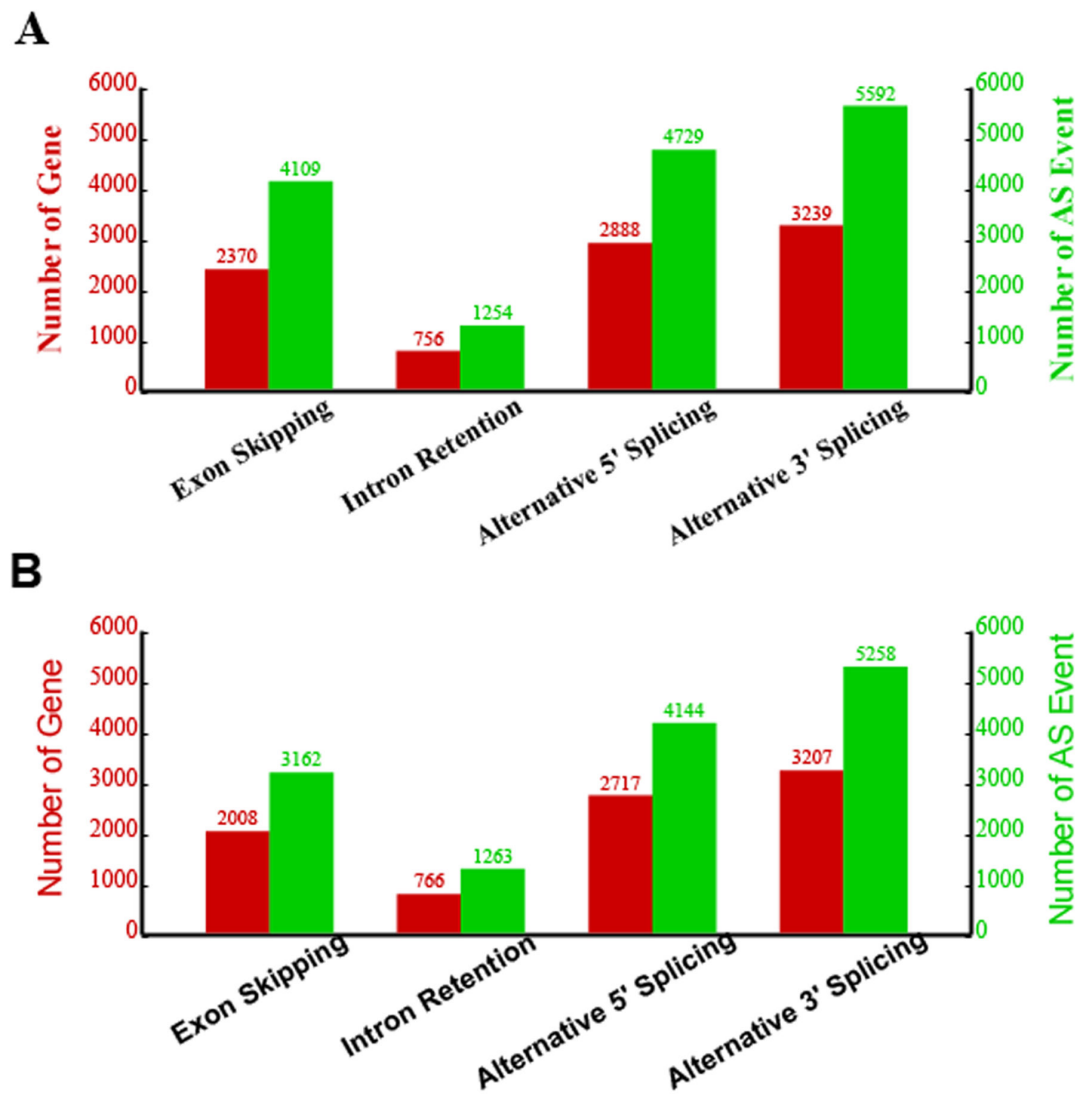
Alternative splicing events and genes detected between the two samples. This diagram depicts the number of AS events, including all four event types in the two samples. (A) shows AS in the DP and (B) shows the same for the SH. The red bars and coordinates represent reference genes subject to AS. The green bars and coordinates show the number of every AS type. A3SS (alternative 3’ splicing) was the principal type of AS found, followed by A5SS (alternative 5’ splicing).

**Figure 8:**
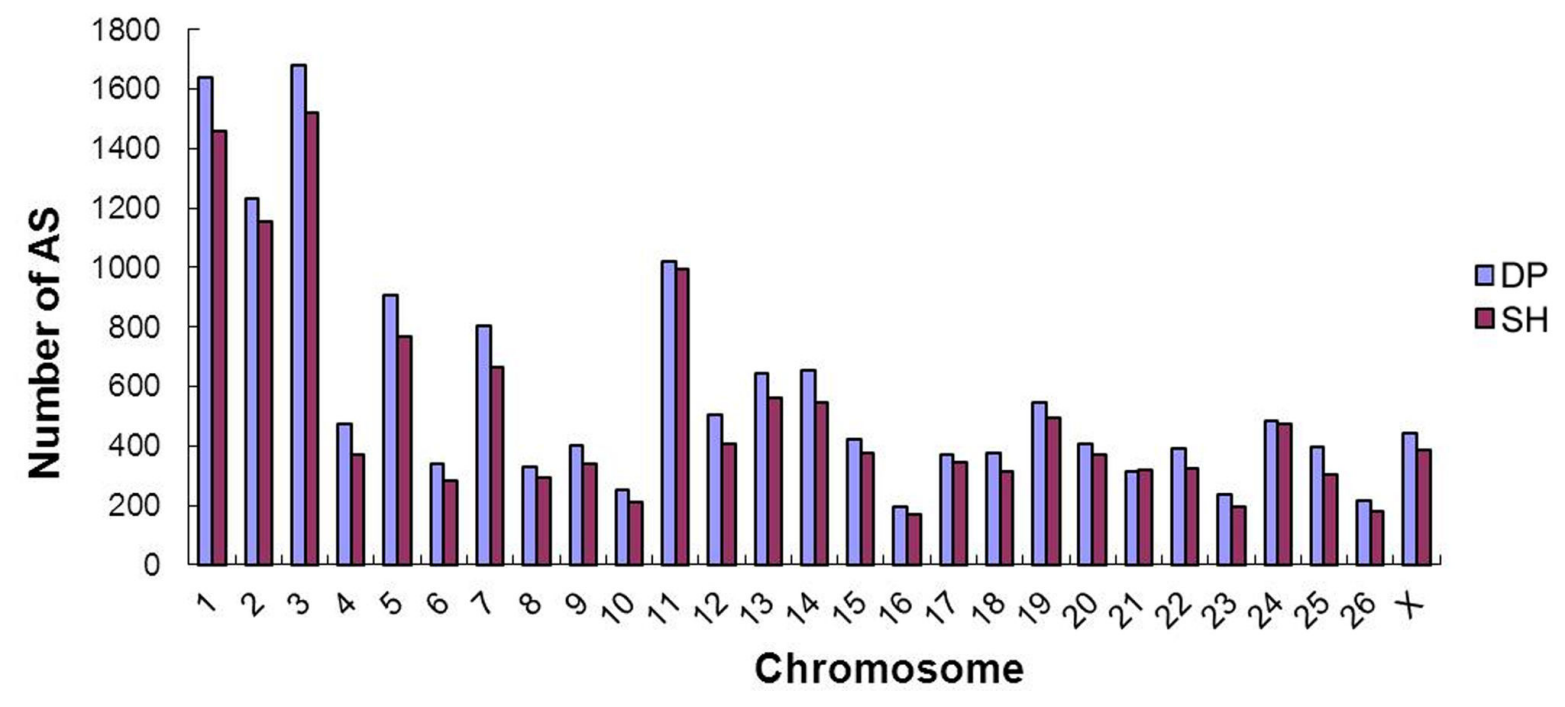
Alternative splicing events on every chromosome. The distribution of the 29,511 putative AS events detected in the two samples on the 26 autosomes and the X allosome of the sheep are shown. All of the AS distributed primarily on chromosomes 1, 2 and 3.

AS is a mechanism that brings remarkable diversity to proteins and allows a gene to generate different mRNA transcripts that are translated into distinct proteins. AS is known to be universal in eukaryotes. Four known types of AS were considered in this study, including alternative 3’ splicing site (A3SS), alternative 5’ splicing site (A5SS), intron retention (IR) and exon skipping (ES). The two libraries had the same rate of AS, with up to 5,265 (26.02%) and 5,116 (25.28%) of the reference genes undergoing 15,684 and 13,827 AS events in the DP and SH libraries, respectively. The distribution of the AS events is shown in Table S5 and summarized in Table 5. The numbers of AS events occurring on the + and - strands of genes were nearly equal in the two samples. Among the four types of AS, A3SS was the most common, accounting for more than one-third of all AS events in the DP and SH transcripts (5,592/15,684 and 5,258/13,827, respectively), followed by A5SS. IR was the least frequent (Figure 7). To delineate an AS-inactive region, we analyzed the number of AS events on every chromosome. As shown in Figure 8, we found that AS primarily occurred on chromosomes 1, 2 and 3. Between the two samples, approximately 20.54% (6,062/29,511) of the AS transcripts were completely in common and appeared to be spliced by all of the four types of AS (Table 6). Further study suggested that 1,264 genes utilized AS in DP but not in SH sample, and 1,114 genes utilized AS in SH but not in DP. Interestingly, we found that the same gene could have used different types of AS both within the sample or between the two samples. Next, we mapped all of the different AS events between the two libraries onto the 27 chromosomes (Figure S2). We found that the two samples had similar distributions and scattered unevenly on each chromosome from 5’ to 3’ and were relatively concentrated in certain regions of the chromosomes. For example, the different AS was clustered on a 40-45 Mb region of the chromosome 21. Between the two libraries, the number of different AS was obvious in some chromosomal regions. For example, there were obviously fewer SH AS in a region of 75 Mb of chromosome 10 than DP AS.

**Table 5:** Alternative splicing (AS) events and their distribution on the + and - strands of the reference genes.

AS type	DP			SH		
	+	-	Total	+	-	Total
A3SS	2,852	2,740	5,592	2,693	2,565	5,258
A5SS	2,379	2,350	4,729	2,069	2,075	4,144
IR	680	574	1,254	693	570	1,263
ES	2,123	1,986	4,109	1,597	1,565	3,162

**Table 6:** A summary of common differences of every AS type between the two samples.

AS type	Gene number	Incompletely repeated AS	Completely repeated AS	Total AS
A3SS	4,292	8,667	2,183	10,850
A5SS	3,870	7,114	1,759	8,873
IR	1,056	1,862	655	2,517
ES	3,088	5,806	1,465	7,271
Total	12,306	23,449	6,062	29,511

### Optimization of Annotated Gene Structures

Extension of the 5’ and 3’ ends of genes is valuable to define the gene’s boundary more precisely, which is helpful for finding genomic loci containing transcripts for further analysis. In this study, we investigated the upstream and downstream regions of all transcripts by comparing the gene models obtained by RNA-seq with existing gene annotations. The extended results are shown in Table S6. We assigned 6,989 reference genes 12,908 different extensions according to the two sets of sequencing data. However, there only 1,376 genes were extended in the DP dataset and 1,731 in the SH dataset. In the optimized results, 1,700 genes were extended at the 5’ end, 3,989 genes at the 3’ end and 1,299 genes at both the 5’ and 3’ ends. There were 1,875 (27.20%) genes extended in at least two regions. However, 4,502 genes were extended in different regions between the two libraries.

## Discussion

Analysis of the global gene expression patterns may provide a comprehensive understanding of transcriptome characteristics. In this study, we provided more comprehensive insight into the ovine muscle transcriptome using deep sequencing. Because of its high throughput, accuracy, repeatability and low signal-to-noise ratio, the Illumina high-throughput sequencing platform is widely used for genome and transcriptome analysis based on the sequencing-by-synthesis (SBS) technique. This efficient deep sequencing not only allows the analysis of transcriptome characteristics but also improves gene annotations at single-nucleotide resolution to provide valuable and complementary data for further studies. Currently, the Illumina high-throughput sequencing is widely used as a powerful tool for the identification of SNPs [30], AS [31], novel genes [32] and other genetic traits in a variety of organisms.

In this study, we took the DP and SH as our studied animals. These sheep species come from different countries and their growth rate is very different. The DP is a South African mutton breed and grows rapidly. However, the SH is a native breed to China with a slow growth rate. The appearance and shape of the two animals we selected from each breed completely conformed to their varietal characteristics and came from two purebred herds, respectively. They were the same age (11 months) and fed in the same way in a farm, but their weight difference was big (SH’s weight was 39 kg and DP was 64 kg). Through comprehensive consideration of the above factors, we think that the selected two sheep had certain representativeness of their own breed. The different growth speed of muscle may be associated with the difference or different regulation of gene expression. The obtained transcriptome sequences provide fundmantal data for further study.

For the two libraries, 9.27 Gb of sequence data were obtained, which is approximately three-fold larger than the sheep genome. The tag density was sufficient for the quantitative analysis of gene expression. A total of 103 Mb reads were obtained from RNA-seq analysis of the two samples, with more than two-thirds of the reads mapped to the reference genome. Compared with the reports of pigs [33] and bovine [34,35], in which 61.4-65.6% of reads mapped to the reference genome, our mapping percentage was not low for a non-model organism. An assessment of the sequencing randomness showed that the reads were distributed evenly over genes from the 5’ to 3’ end, which confirmed that the library preparation and sequencing in our study was successful. The percentage of reads that mapped to the sheep genome in the DP (71.82%) was slightly higher than for the SH (68.05%), but the percentage of mapped reads to reference genes was not similar. The unmatched read mapping indicated that gene expression was different between the two samples. It should be mentioned that the total percentages of the unmapped reads were too high when mapped to the reference genes (57.23% and 66.90%, respectively). This result might have been caused by imperfections in the reference genome, reference errors, sequencing errors and the chosen mapping criteria.

RPKM fold changes may be used to characterize gene expression levels [31,35]. In this study, a majority of the annotated genes (>62%) in the sheep genome database was covered by more than 80% of the sequencing reads, showing the sensitivity of RNA-seq in transcript discovery even for lowly-expressed genes, similar to evidence reported by Wang (2009) [20]. The correlation between gene expression levels of the samples was very high (r = 99.98%), which suggested that a large fraction of the transcripts was conserved across individuals. These results were consistent with those observed in other mammalian species with RNA-seq [30,36].

RNA-seq provides a more sensitive platform for measuring differentially expressed genes than traditional microarray hybridization [37]. Several studies have identified molecular mechanisms in sheep muscles using microarrays [11,38]. However, to our knowledge, very few articles have addressed the DEGs of ovine muscle using the next-generation sequencing. Here, we presented a preliminary analysis of differentially expressed muscle genes between DP and SH. In this study, 1,300 DEGs with 24 and 17 highly expressed genes in the DP and SH libraries, respectively, were found. Regarding the growth characteristics of the two sheep, those highly expressed DEGs will be the focus of our further studies.

Our sequencing data profoundly improves the existing gene annotation. As has been reported in rice [28] and pigs [33], we found numerous novel TUs. These data will contribute to finding novel genes and perfecting the current sheep genome annotations.

SNPs have been increasingly employed due to their high number, low cost and ease of scoring. In addition, SNPs can be found inside candidate genes for artificial or natural selection and might therefore be more informative than other molecular markers. Next-generation sequencing has been recently been applied to analyze SNPs in eukaryotes [13,14,39]. In this study, we ovserved that the two samples had similar base changes, with the most common being A/G, followed by T/C and C/T. However, other types of nucleotides changes showed slight differences, and the third most common change was G/R in SH and C/T in DP. When all observed or different cSNPs were mapped to chromosomes, there was little difference in distribution between the two libraries. The two samples’ inconsistencies suggested that gene expression and regulation might be very different between the two sheep. Because chromosomes 1, 2, and 3 are longer than the other chromosomes, the number of cSNPs that mapped to these chromosomes was higher than for the other chromosomes. In general, cSNPs will be valuable molecular markers for further study and can act as candidate markers for identification of eQTL traits.

Another important advantage of RNA-seq is its ability to detect AS events. AS is an important model of gene expression regulation and is not generally assessable using microarray or Sage methods. In the two samples, we found that they had similar AS rates with more than one-fourth (26.02% and 25.28%, respectively) of the reference genes undergoing 29,511 AS events. This percentage was much lower than the 86.0% reported for humans [26] and 33.0% for rice [28] but was much higher than the 18.0% reported for pigs [33]. In addition, we found that A3SS was the most common type in sheep. This is similar to pigs [33] but different from rice, in which intron retention is the primary AS type [28]. This result is also different from the reported AS events in humans [12] and yeast [15], in which exon-skipping is the most prevalent mechanism. Expression analysis has showed that AS events in the two samples occurred actively on chromosomes 1, 2 and 3, which demonstrated that the frequency of AS events was consistent with the sheep chromosome length (chromosomes 1, 2 and 3 are the longest). However, the AS numbers in DP were higher than SH, which is in contrast with the cSNPs. The contrary result suggests that same gene might undergo different transcriptional and translational regulation in the two animals. Further research will be able to determine the detailed regulation in each breed, and these observed differences observed will be highly useful in future studies.

## Conclusions

This study describes the first muscle transcriptomes of the DP and SH using deep sequencing on the Illumina HiSeq^TM^ 2000 high-throughput platform. In summary, we obtained two libraries of 50,264,608 and 52,794,216 high-quality reads for SH and DP, respectively. In addition, we identified and compared important transcriptome features, such as AS event and cSNPs. We extended 6,989 reference genes, and 123,678 novel transcript units were identified from the two datasets. We found that 1,300 genes were significantly differentially expressed between the two libraries. All of these results suggested that there were many differences in the muscle transcriptomes between the two individuals. These sequencing data and analyses will provide fundamental information for future studies.

## Supporting Information

Figure S1 **Comparative analysis of all differential cSNPs between the two samples on 27 (26 autosomes and X allosome) ovine chromosomes.** Each chromosome is represented along the X-axis and binned into windows with 100,000 bp/window. The Y-axis shows cSNPs counts. The red line indicates different cSNP in DP, and the green line indicates different cSNPs in SH.
(TIF)

Figure S2 **Comparative analysis of all differential AS events between the two samples on 27 ovine chromosomes.** Each chromosome is represented along the X-axis and binned into windows with 100,000 bp/window. The Y-axis shows AS counts. The red line indicates different AS in DP, and the green line indicates different AS in SH.
(TIF)

Table S1 Expression statistics for reference genes in the Dorper (DP) and Small-tailed Han (SH) ovine transcriptomes.
(XLSX)

Table S2 Differentially expressed genes between the DP and SH transcriptomes.
(XLS)

Table S3 Novel transcript units in the DP and SH transcriptomes.
(XLSX)

Table S4 Distribution of cSNPs in the SH and DP compared with the *Ovis aries* genome.
(XLSX)

Table S5 AS statistics for the DP and SH.
(XLSX)

Table S6 **Gene extension according to the transcripts from the DP and SH.**
(XLSX)
