# Supplementary figures and images for: Characterization and Comparative Analyses of Muscle Transcriptomes in Dorper and Small-Tailed Han Sheep Using RNA-Seq Technique
Source: PLoS One. 2013 Aug 30;8(8):e72686. doi: 10.1371/journal.pone.0072686 (PMC3758325; doi:10.1371/journal.pone.0072686)

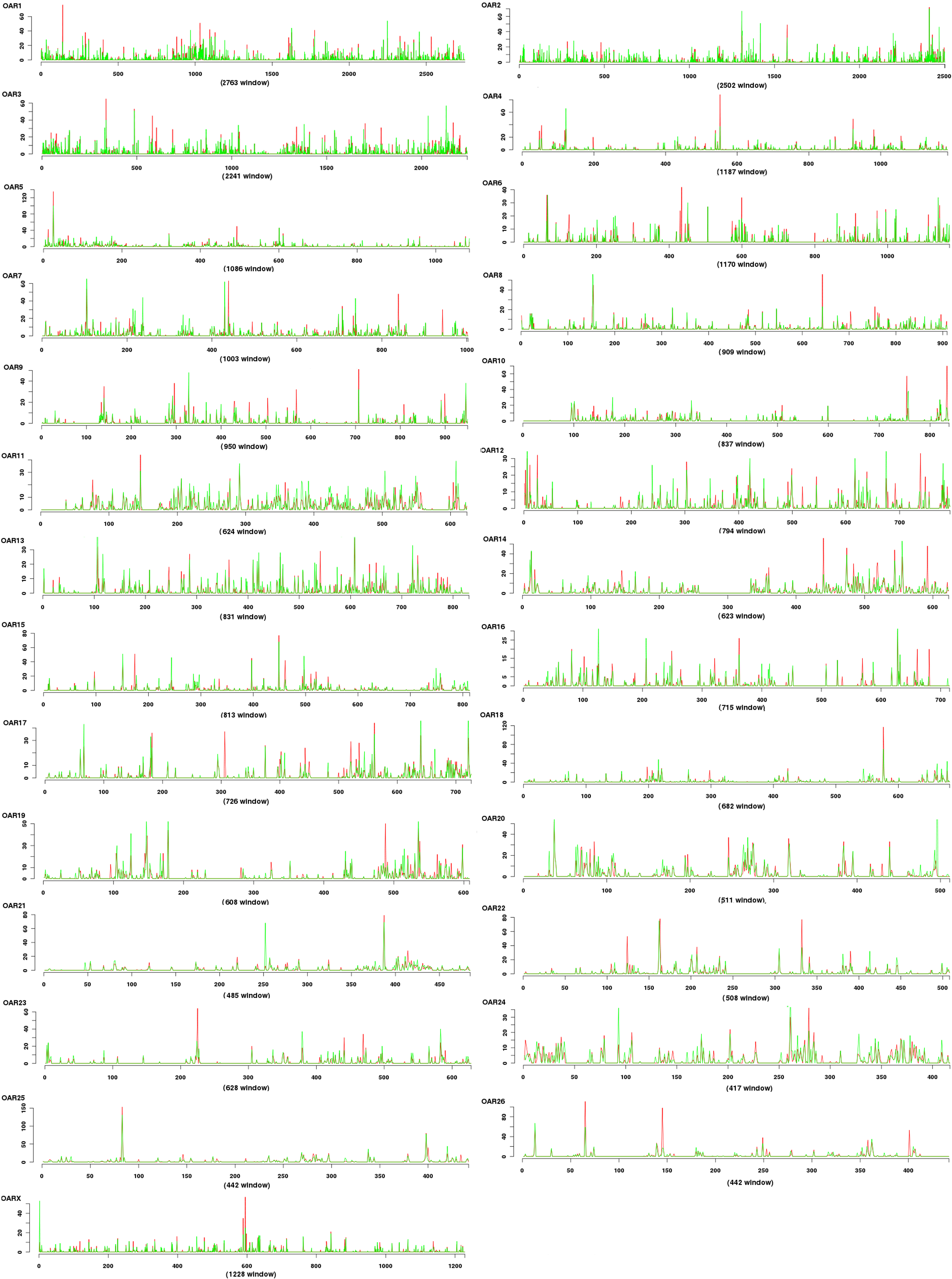

Supplement: Figure S1 — Comparative analysis of all differential cSNPs between the two samples on 27 (26 autosomes and X allosome) ovine chromosomes. Each chromosome is represented along the X-axis and binned into windows with 100,000 bp/window. The Y-axis shows cSNPs counts. The red line indicates different cSNP in DP, and the green line indicates different cSNPs in SH. (TIF) [file pone.0072686.s001.tif]

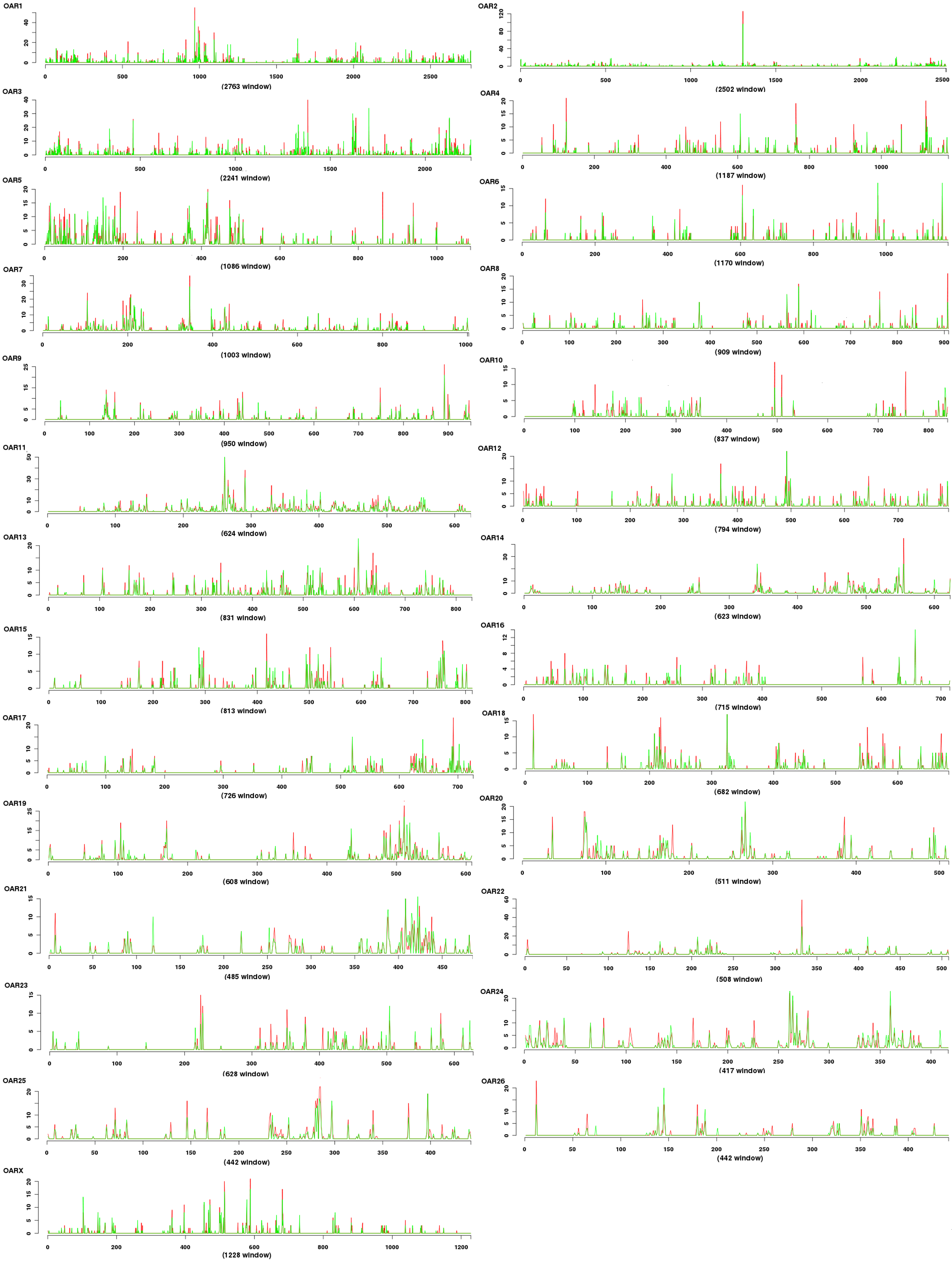

Supplement: Figure S2 — Comparative analysis of all differential AS events between the two samples on 27 ovine chromosomes. Each chromosome is represented along the X-axis and binned into windows with 100,000 bp/window. The Y-axis shows AS counts. The red line indicates different AS in DP, and the green line indicates different AS in SH. (TIF) [file pone.0072686.s002.tif]
